# Three Experimental Common High-Risk Procedures: Emission Characteristics Identification and Source Intensity Estimation in Biosafety Laboratory

**DOI:** 10.3390/ijerph20054479

**Published:** 2023-03-02

**Authors:** Zhijian Liu, Jiabin Lv, Zheng Zhang, Juntao Ma, Yangfan Song, Minnan Wu, Guoqing Cao, Jiacheng Guo

**Affiliations:** 1 Department of Power Engineering, North China Electric Power University, Baoding 071003, China; 2 Institute of Building Environment and Energy, China Academy of Building Research, Beijing 100013, China

**Keywords:** bioaerosol, risk assessment, gaussian mixture model, quantitative analysis

## Abstract

Biosafety laboratory is an important place to study high-risk microbes. In biosafety laboratories, with the outbreak of infectious diseases such as COVID-19, experimental activities have become increasingly frequent, and the risk of exposure to bioaerosols has increased. To explore the exposure risk of biosafety laboratories, the intensity and emission characteristics of laboratory risk factors were investigated. In this study, high-risk microbe samples were substituted with Serratia marcescens as the model bacteria. The resulting concentration and particle size segregation of the bioaerosol produced by three experimental procedures (spill, injection, and sample drop) were monitored, and the emission sources’ intensity were quantitatively analyzed. The results showed that the aerosol concentration produced by injection and sample drop was $10^{3}$ CFU/m${}^{3}$, and that by sample spill was $10^{2}$ CFU/m${}^{3}$. The particle size of bioaerosol is mainly segregated in the range of 3.3–4.7 μm. There are significant differences in the influence of risk factors on source intensity. The intensity of sample spill, injection, and sample drop source is 3.6 CFU/s, 78.2 CFU/s, and 664 CFU/s. This study could provide suggestions for risk assessment of experimental operation procedures and experimental personnel protection.

## 1. Introduction

The frequent occurrence of infectious diseases such as novel coronavirus and SARS-CoV-2 has seriously affected human health and the social economy [1,2,3,4]. There are numerous reports on the transmission of infectious microbes in the form of aerosols [5,6,7]. Biosafety laboratories have a set of preventive measures required for handling dangerous biological agents in a safe [8], reliable, and closed environment, and is the main location for studying unknown microbes [9]. However, in the course of the research, due to accidents or the carelessness of operators [10], highly contagious microbes will spread to the surrounding environment in the form of aerosols [11], presenting a large exposure risk to researchers. Research has shown that 86.6% of operations can cause both microbe aerosols and unexplained laboratory infections that may be caused by the diffusion of microbe aerosols in the air, according to 276 types of operational testing in laboratories. Therefore, detailed information on the characteristics of aerosols in different experimental procedures is essential for assessing human health and the environment, as well as the source identification and apportionment of these particles.

Risk assessment is the process of evaluating the risks associated with working with hazards. Factors such as the quantity and concentration of infectious substances, pathogenicity of biological agents, and possibility of aerosol generation in the working process should be considered in the evaluation process [3,12]. A quantitative analysis of bioaerosol concentration produced by centrifuge centrifugation and freeze-dried powder being dropped in biosafety laboratories has been reported [13]. To further reduce the risk of assessment, many studies chose Serratia marcescens, which is harmless to the human body, as a substitute for quantitative analysis for reduction experiment simulations [14]. Zhuang et al. [15] used Serratia marcescens as the model bacteria for experimental verification. Based on this, they used a numerical simulation method to explore the spatial transport and deposition behavior of a biosafety laboratory pollution source after leakage and determined the most serious pollution location in the vortex area and high-concentration area of pollutants. Long et al. [16] carried out a biosafety risk assessment and control of laboratory tests, except for nucleic acid tests, in the clinical laboratory of a COVID-19-designated hospital, emphasizing the high risk of bioaerosol transmission. Wen [17] used Serratia marcescens in a biosafety laboratory as a replacement for high-risk microbes and simulated experimental operations such as pipetting and high-speed centrifugation. The aerosol concentration generated in each experimental activity was quantitatively analyzed. Li et al. [18] summarized the risk factors for the generation of bioaerosols in the experimental activities, where sample spill, sample drop, and injection were among the high-risk factors. However, there are few reports on a biosafety assessment based on the particle size segregation and source characteristics of bioaerosols produced by high-risk factors in advanced biosafety laboratories.

In a laboratory environmental risk assessment, the source of pathogenic microorganisms is the decisive factor [19,20]. Afshari detected 13 different particle sources in a 32 m${}^{3}$ full-size chamber and quantified the emission of ultrafine and fine particles 22 for the first time [21]. Clemente [22] simulated the release of hazardous nanoparticle material in a specially designed 13 m${}^{3}$ stainless steel vessel under accidental conditions. Many studies have designed facilities to study and verify the indoor environment in which the treated material is released [23]. However, none of these studies have addressed the issue of source-released aerosols. Studies have pointed out that the study of source intensity plays an important role in reducing air pollution [24,25]. Mei [26] established a probability model based on Markov chains to simulate the transport and diffusion of air pollutants released by pollution sources, but the model did not consider the effect of temperature and humidity on the diffusion of pollutants. A Gaussian mixture model is often used to study the source intensity and spatial concentration of atmospheric pollutant emissions [27]. Compared with other models, the temperature and humidity are included in the independent variables of the model, which further improves the accuracy of the spatial concentration prediction of biological aerosols [28,29,30,31]. However, there are few reports on the risk assessment of experimental operations from the source direction, and few studies have included aerosol source strength in the risk assessment.

In this study, the characteristics of aerosol pollution release sources and the spatial distribution of the concentration affected by typical risk factors such as sample spill, injection, and sample drop were investigated through experiments in an exposure chamber. Concentration-monitoring experiments of different risk factors with time were carried out in this chamber. The main work and contributions are as follows: (1) three common high-risk factors, such as sample spill, injection, and sample drop, were experimentally reduced in a small chamber, and the concentration and particle size segregation of bioaerosols produced by them were sampled and monitored; (2) a quantitative analysis of the relationship between risk factors, concentration, and particle size segregation, and a determination of the characteristics of each risk factor release source, were carried out; (3) a Gaussian mixture model was used to calculate the pollution source intensity of each hazard factor; (4) a risk assessment of high-risk factors of biosafety laboratories was conducted from the source, and effective protection suggestions were put forward that provided a reference for the risk prediction.

## 2. Method

### 2.1. Experimental Method

#### 2.1.1. Measurement Instruments

Aerosol sampling

Bioaerosol samples are collected using the Anderson six-stage sampler, which is a common sampling methods [32]. The sampling device consists of 6 stages according to the dynamic diameter: 0.65–1.1 μm, 1.1–2.1 μm, 2.1–3.3 μm, 3.3–4.7 μm, 4.7–7.0 μm, and >7.0 μm [33]. Aerosol samples were collected using LB Nutrition Agar and incubated at 37 ${}^{\circ }$C for 24 h, then cultured at 27 ${}^{\circ }$C for 24 h until colonies turned bright red. After, they were counted [34].

When sampling, the superposition of multiple colonies may occur in a sampling well, resulting in deviation in counting. Therefore, we performed a positive hole correction [35]. The method used for calculating the bioaerosol concentration is as follows:

(1) $$P_{r}=N\times \left(\frac{1}{N}+\frac{1}{N-1}+\frac{1}{N-2}+\cdots +\frac{1}{N-r-1}\right)$$

(2) $$C_{a}=\frac{P_{r}}{Q\times T}\times 1000$$

where $P_{r}$ is the corrected colony number; r is the actual number of colonies; *N* Number of holes at each level of the sampler, *N* = 400; $C_{a}$ is aerosol concentration, $P_{r}$ is the sum of colony number corrected on six Petri dishes; *Q* is the collection flow rate 28.3 mL/min; *T* is the sampling time, min.

In this study, impacted bacteria were sampled, and the austenitic transformation method was used to change the concentration of sedimentation bacteria. The method was used to investigate the amount of bacteria deposited on a 10 cm medium surface in 5 min, which was equivalent to 10 L of air [36]. The formula is as follows:

(3) $$C=\frac{100}{A}\times \frac{5}{t}\times \frac{1000}{10}\times N$$

where *C* is the number of airborne aerosols, CFU/m${}^{3}$; *A* is the area of the plate used, cm${}^{2}$; *t* is the exposure time of the plate, min; *N* is the number of the colony on the plate, CFU.

Particle sampling

In this study, TSI (3330) was used to monitor the released particles, the sampling flow was 10 L/min, and the particle sizes of the 16 sampling channels were 0.3 /0.4 /0.5 /0.6 /0.7 /0.9 /1.1 /1.4 /1.7 /2.1 /2.7 /3.3 /4.1 /5.2 /6.5 /8.1 /10 μm.

#### 2.1.2. Material

Serratia marcescens is a model bacterium commonly used in laboratories [18]. Its diameter is approximately 0.5–0.8 μm it causes very little harm to humans and animals, it has no spores, and it is easy to distinguish from other miscellaneous bacteria. In addition, the strain used in this study can produce blood pigment—prodigiosin—for easy tracking and identification [37,38]. Serratia marcescens was stored at −80 °C, and 10 mL of the microorganism was activated in 20 mL of Nutrient Broth at 37 °C and 250 rpm; after 24 h, media were striated in Nutrient Agar plates and incubated at 28 °C for 24 h. A single colony was taken and inoculated into the Nutrient Broth and cultured at 37 °C. A standard inoculum of 1 × $10^{9}$ CFU/mL was established using NB and kept at −80 °C [37]. Nutrient AGAR composition: peptone 1%, beef extract 0.3%, sodium chloride 0.5%, AGAR 1.5–2.0%, distilled water preparation. pH 7.2–7.4. Nutritious broth composition: peptone 1%, beef extract 0.3%, sodium chloride 0.5%, distilled water preparation, pH 7.2–7.4 [37].

#### 2.1.3. Experimental Design

In this study, three high-risk experimental factors were investigated: (I) Sample spill: the operator used a pipette to aspirate and mix 30 mL of concentrated bacterial solution. When pipetting bacterial solution, the operator must avoid the formation of bubbles and splashes. Pipetting was performed every 5 s for 5 min; (II) Injection: the operator injected a syringe containing 2 mL of bacterial solution into the air once every minute, 5 times in total. This operation is used to simulate an accidental jetting resulting from an animal injection; (III) Sample drop: a conical flask containing 30 mL of bacteria was dropped from 1.2 m at 45° to the ground.

**Figure 1:**
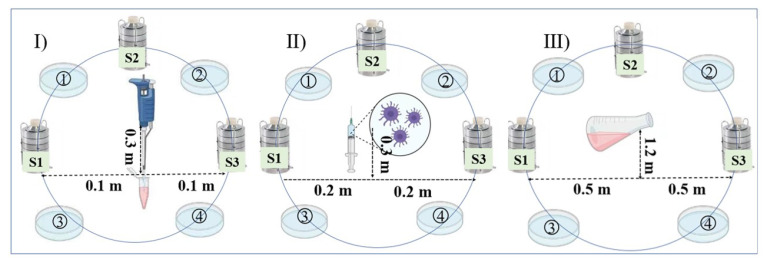
Sampling point location.

**Figure 2:**
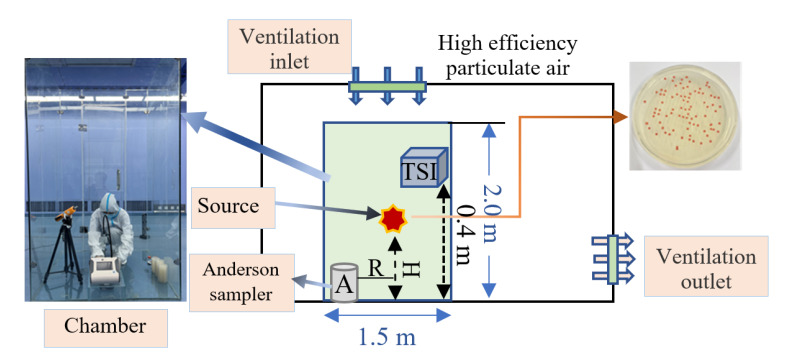
Experiment site and environment.

Air samples were collected for 10 min with an Anderson six-stage sampler, which was used at a flow rate of 28.3 L/min (Figure 1) [39,40,41]. Samples obtained from the Anderson six-stage sampler were taken to the laboratory for culture less than 4 h after sampling. The particle counter was sampled at 40 cm on the vertical ground, as shown in Figure 2, and measured every minute. The above experiments were repeated 6 times for each group.

This study was carried out in a glass chamber with dimensions of 1.5 m × 1.5 m × 2.0 m (corresponding, to length × width × height, respectively) (Figure 2). A dispersal chamber is a qualified chamber with a tightly sealed door and walls, with a specified inflow of high-efficiency particulate air and filtered air and a controlled outflow. During the experiment, the clean room temperature was set at 26.0 °C and the relative humidity was set at 50% [41]. The environmental parameters of the small room were monitored as shown in Table 1. The ventilation system can effectively keep the experimental environmental parameters stable. In addition, the vertical ventilation system can quickly eliminate aerosols in the chamber. Before the experiment, ultraviolet lamp sterilization and alcohol wiping were used for disinfection each time. Anderson’s six-stage sampler was sampled for 10 min before each experiment as a blank control.

### 2.2. Mathematical Method

#### 2.2.1. Source Intensity

The source intensity (Q) is defined as the number of bacteria released by the infectious source per second (CFU/s) [24]. The Gaussian mixture model is commonly used to calculate the diffusion concentration of pollutants continuously discharged into the air from a point source [42]. In this study, the diffusion concentration was measured experimentally. The Gaussian mixture model retrieved the aerosol source intensity [43]. The establishment of the Gaussian diffusion model is based on four assumptions: 1. Uniform, stable, and continuous discharge of pollution point sources; 2. Air pollutants follow the conservation of mass in the diffusion process; 3. The wind direction in the diffusion area is uniform and stable; 4. The pollutant concentration is in accordance with normal distribution in the horizontal direction and normal distribution in the vertical direction. The core principle formula is as follows:

(4) $$C_{\left(x,y,z\right)}=\frac{Q}{2\pi u\delta_{y}\delta_{z}}\exp\left[-0.5{\left(\frac{y}{\delta_{y}}\right)}^{2}\right]\cdot \left\{\exp\left[-0.5{\left(\frac{z-H}{\delta_{z}}\right)}^{2}\right]+\exp\left[-0.5{\left(\frac{z+H}{\delta_{z}}\right)}^{2}\right]\right.$$

where *Q* is pollutant discharge rate per unit time, CFU/s; *H* is the effective height of the pollution source, m; *u* is the average wind speed at the source of pollution, m; *y* is the horizontal coordinate perpendicular to the *x*-axis, m; *z* is the vertical coordinates, m; $\delta_{y}$, $\delta_{z}$, diffusion coefficients in horizontal (y) and vertical (z) directions, m.

#### 2.2.2. Data Statistics

In this study, we conducted three groups of parallel control experiments for each group of experiments, proofread the collected data using the 3-sigma method, and calibrated and rejected data with large errors. This study used SPSS 25.0 for statistical analysis. *p* < 0.05 is statistically significant, and all tests were two-tailed. In addition, one-way analysis of variance tests were used. For the data and continuous variables that did not conform to normal distributions, the non-parametric Wilcoxon rank sum test was used to test the aerosol-emission-related factors according to their distribution.

## 3. Results

### 3.1. Aerosol Emission Level

In this study, an experimental operation of three risk factors was simulated, and emission characteristics of bioaerosol sources were quantitatively analyzed.

**Figure 3:**
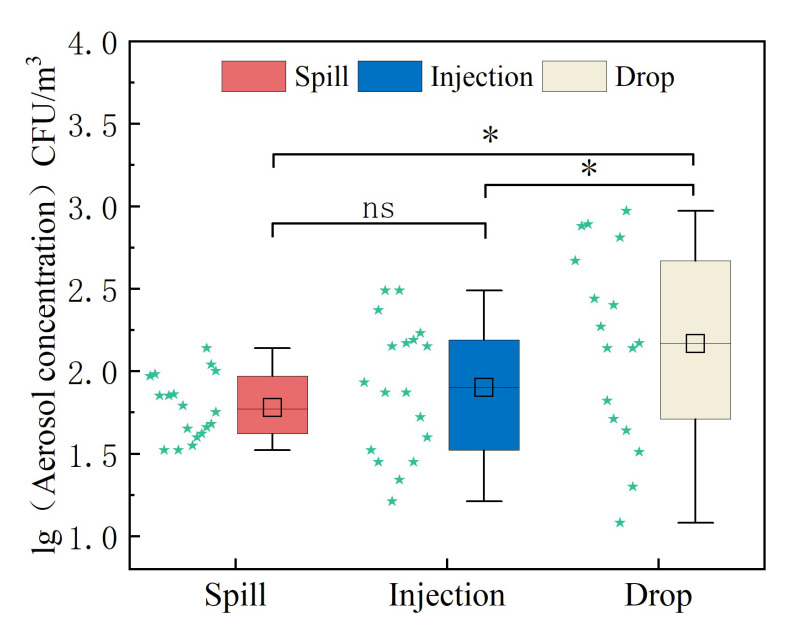
Relationship between aerosol concentration and experimental risk factors. * *p* < 0.05, the statistically significant difference in aerosol concentration among the three risk factors; ns, no statistical difference (one-way ANOVA test).

As shown in Figure 3, the concentration range of aerosols formed by the sample spill is 10–$10^{2}$ CFU/m${}^{3}$, the total aerosol generated by the injection is $10^{2}$–10${}^{3}$ CFU/m${}^{3}$, and the aerosol distribution generated by the drop is 10–$10^{3}$ CFU/m${}^{3}$. The three groups of samples conform to the normal distribution after taking logarithms. The single-factor analysis of variance shows that the aerosol concentrations were statistically different between the accidental drop of conical bottles and the other two experimental groups, whereas the aerosol concentrations were not statistically significant between the accidental leakage of the spill and the injection. The average aerosol concentration generated by the sample drop was 1108.2 CFU/m${}^{3}$, and the upper quartile was 1808 CFU/m${}^{3}$. The average concentration was 78.3 CFU/m${}^{3}$, and the quartile was 988.4 CFU/m${}^{3}$. The aerosol concentration produced by the suction of a high concentration of bacterial liquid was 250.3 CFU/m${}^{3}$, and the upper quartile was 485.2 CFU/m${}^{3}$. The concentration of the bioaerosol produced by sample dropping was statistically significant (*p* < 0.05), whereas the concentration of the bacterial liquid had no statistical significance regarding the concentration of the aerosol produced by the two risk factors.

**Figure 4:**
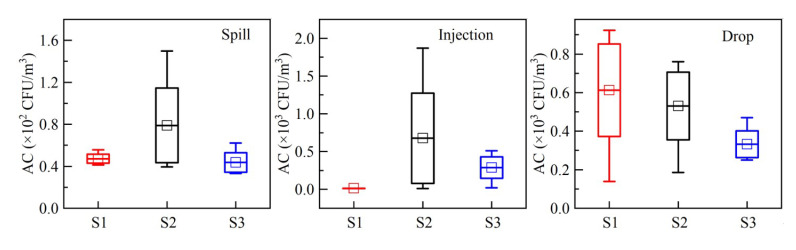
Culturable bioaerosol concentration at each sampling point. AC is aerosol concentration.

In this study, by monitoring the three sampling points in each experiment operation, it was found that the concentration of the bioaerosol located in front of the operator has obvious advantages compared to when it is located on either side, see Figure 4. The shape of the injection and sample spill is the main flow type, and the main flow direction varies according to the operator’s practice. According to the assumption of the Gaussian mixture model application, we regarded the direction of the maximum concentration as the wind direction in the model.

**Figure 5:**
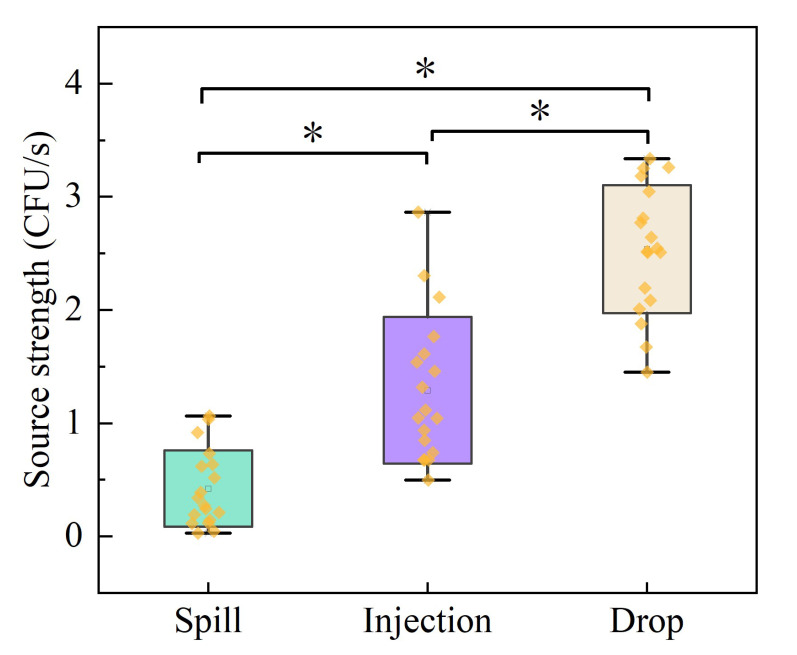
Aerosol release source intensity. * *p* < 0.05, Statistical significance between source intensity of three risk factors, ns is no statistical difference. (Wilcoxon Paired Sign Rank Test).

The source intensity is defined as the emission rate of pollutants. In this study, a Gaussian mixture model was used to inversely calculate the source intensity of the three test factors, as shown in the table. The source intensity of the spill factor was 3.6 CFU/s, the source intensity of the injection was 78.2 CFU/s, and the source intensity of the aerosol caused by the sample drop was 664.1 CFU/s. There were significant differences in the aerosol source intensity caused by the three risk factors (*p* < 0.05), as shown in Figure 5. The sample spill, injection, and sample drop sources differ by order of magnitude, which may be related to the volume of bacteria released into the air during each high-risk operation.

### 3.2. Bioaerosol and Particle Size Segregation

**Figure 6:**
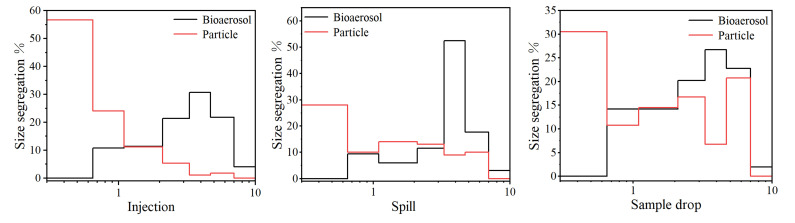
Mass concentration segregation.

We analyzed the size of the culturable bioaerosol collected. As shown in Figure 6, the bioaerosol size distribution of culturable fungi produced by the three experimental schemes presented an n-type, with the particle size mainly distributed in the range of 3.3–4.7 μm, accounting for 54.6% (sample spill), 32.6% (injection), and 27.8% (sample drop) of the total amount of emissions, respectively. With 3.3–4.7 μm as the center, the number of aerosols decreased gradually with a change in the particle size. In addition, the main bioaerosol sizes of the aerosol produced by the injection were 2.1–3.3 μm (22.7%), 3.3–4.7 μm (32.1%), and 4.7–7.0 μm (23.5%), respectively. The main particle size ranges of the aerosol in the sample drop were 2.1–3.3 μm (21.7%), 3.3–4.7 μm (27.6%), and 4.7–7.0 μm (24.9%).

The main particle size range produced by each experimental operation was 0.3–0.65 μm, accounting for 28.9% of the total emission (sample spill), 56.4% (injection), and 31.9% (sample drop), respectively. The size of particles in the range of 0.65–7.0 μm produced by the sample spill and injection was less than 20% of the total monitored size, and the number of particles tended to decrease with the increase in particle size. The particle size segregation generated by the sample drop was roughly consistent with that of aerosol, and the particle size mainly ranged from 4.7 to 7.0 μm.

**Figure 7:**
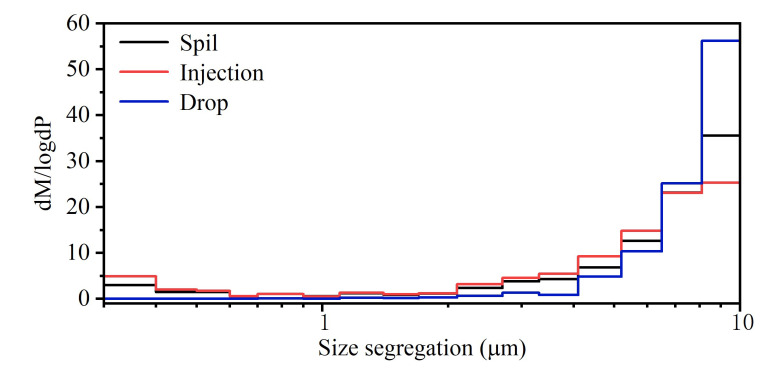
Mass concentration segregation.

Grain size distributions are traditionally described by the sums of several lognormal distributions. The lognormal distribution of the particle mass and particle number was obtained by measuring the three groups of experiments, as shown in Figure 7. The logarithmic normal distribution of the overall mass of the particles released from the experimental activity is u-shaped. The mass of the particle is high at both endpoints and low at the middle point. With an increase in the particle size, the growth trend also gradually accelerates. The number of particles dropped from the sample increased exponentially, and the number of small particles was lower than that of spill.

## 4. Discussion

The experimental activities of the biosafety laboratory mainly involve sample collection, transportation, reception, processing, experimental operation and preservation, waste disposal, and other activities [18]. If the control method is improper, there is a risk that pathogens may infect the laboratory staff or spread to the social population outside the laboratory. The risks of experimental activities vary. A risk assessment of experimental activities, identification of risk sources, and corresponding personal protection measures taken to avoid accidental injury and contact with pathogenic microorganisms are necessary to ensure the safety of experimental personnel [44]. The complexity of the laboratory risk assessment and risk control activities depends on the actual hazard characteristics of laboratory activities. A risk assessment and risk control activities should be carried out according to the characteristics and intensity of the risk sources [45].

Using a Gaussian mixture model, the source intensity was calculated by solving the equations of the aerosol concentration at the three positions measured. It was discovered that the aerosol concentration generated by an accidental injection is approximately $10^{4}$ CFU/m${}^{3}$, the aerosol concentration generated by an accidental drop of culture bottles and accidental overflow of freeze-dried powder is approximately $10^{3}$ CFU/m${}^{3}$, and the aerosol concentration generated by a centrifugal tube rupture and ultrasonic cracking in the process of blowing and suction is approximately 10–100 CFU/m${}^{3}$ [18]. The results of this laboratory research are consistent; thus, special attention should be paid to the bioaerosols generated by various experimental activities and accidents in the laboratory.

The results of this study show that the source intensity of culturable bacteria produced by a spill, injection, and dropping gradually increased, and that there were significant differences between source intensities of bioaerosols produced by the three experimental operations (*p* < 0.05). The reason for this may be that the perturbation effect of the experimental operation on the bacterial solution increases with a change in the intensity. In this study, for example, falling conical bottles resulted in more droplets spilling out of the bacteria than the spill. Droplets that splash into the air carry bacteria, which then aerosol nucleation to form bioaerosols. This leads to an increase in the number of aerosols detected in the air.

In this study, the main aerosol size range measured by the Anderson six-stage sampler was 3.3–4.7 μm, and the highest number of viable bacteria of infectious diseases was in the size range of 2.1–4.7 μm [9]. It is easy for aerosols smaller than 5 μm to enter human lungs, causing harm to the human body [46]. The main particle size range measured by the optical particle counter was 0.30–0.65 μm. The Anderson six-stage sampler cannot pick up these tiny particles (0.3–0.65 μm). Meanwhile, Serratia marcescens aerosols are mainly distributed in the size range of 3.3–4.7 μm, so there were almost no Serratia marcescens aerosols on these 0.3–0.65 μm particles. In general, the size of infectious microorganisms ranges from 0.02–0.30 μm for viruses. Particle sizes of 0.3–0.65 μm can become carriers of viruses, presenting an exposure risk to workers.

Overall, when conducting experimental activities in the biosafety laboratory, the risk of microbial aerosol infection needs to be highly valued, good experimental habits should be maintained, actions such as touching the face should be reduced, and the risk of secondary contact infection caused by microorganisms adhering to the surfaces of laboratory clothes and gloves should be avoided. Personal protection should be carried out well, and the use of respiratory protective equipment in high-risk laboratories should be tested. Appropriate airflow organization should be adopted to quickly remove the carrier particles through the airflow to minimize or eliminate the infection caused by experimental activities and accidents.

## 5. Conclusions

To explore the relationship between risk factors, concentration, and particle size segregation, and to determine the characteristics of a sample spill, injection, and drop release source, experimental operations were conducted in the exposure room. Furthermore, the pollution source intensity of each risk factor was calculated by using a Gaussian mixture model. This study provides a reference for risk prediction during an experiment. The following conclusions are drawn:

1. There were significant differences in the aerosol release source intensities during common high-risk experimental procedures. The source intensities of the sample spill, injection, and drop were 3.6 CFU/s, 78.2 CFU/s, and 664 CFU/s, respectively.

2. The measurable culturable aerosol size segregation is mainly within the range of 3.3–4.7 μm. At the same time, the sample spill and injection can produce a large number of particles with sizes ranging from 0.3–0.65 μm.

3. It is recommended to strengthen the elimination of aerosols generated by experimental operations, especially those that can produce fine particles, and to select an effective air distribution.

## Figures and Tables

**Table 1:** Environmental parameters in the sampling process.

	Experimental Preparation	Blank Control Experiment	Formal Experiment	Disinfection Sterilization
Temperature (${}^{\circ }$C) Mean ± SD	26.1 ± 0.27	26.1 ± 0.27	26.1 ± 0.27	26.1 ± 0.27
Relative humidity (%) Mean ± SD	50.3 ± 0.013	50.3 ± 0.013	50.4 ± 0.013	50.5 ± 0.013
Airspeed (m/s) Mean ± SD	0.10 ± 0.135	0.03 ± 0.002	0.03 ± 0.002	0.10 ± 0.135

## Data Availability

The data that support the findings of this study are available from the corresponding author upon reasonable request.
